# Transformation and patterning of supermicelles using dynamic holographic assembly

**DOI:** 10.1038/ncomms10009

**Published:** 2015-12-02

**Authors:** Oliver E.C. Gould, Huibin Qiu, David J. Lunn, John Rowden, Robert L. Harniman, Zachary M. Hudson, Mitchell A. Winnik, Mervyn J. Miles, Ian Manners

**Affiliations:** 1 Bristol Centre for Functional Nanomaterials, HH Wills Physics Laboratory, University of Bristol, Bristol BS8 1TL, UK; 2 School of Chemistry, University of Bristol, Bristol BS8 1TS, UK; 3 School of Physics, University of Bristol, Bristol BS8 1TL, UK; 4 Department of Chemistry, University of Toronto, Toronto, Ontario, Canada M5S 3H6

## Abstract

Although the solution self-assembly of block copolymers has enabled the fabrication of a broad range of complex, functional nanostructures, their precise manipulation and patterning remain a key challenge. Here we demonstrate that spherical and linear supermicelles, supramolecular structures held together by non-covalent solvophobic and coordination interactions and formed by the hierarchical self-assembly of block copolymer micelle and block comicelle precursors, can be manipulated, transformed and patterned with mediation by dynamic holographic assembly (optical tweezers). This allows the creation of new and stable soft-matter superstructures far from equilibrium. For example, individual spherical supermicelles can be optically held in close proximity and photocrosslinked through controlled coronal chemistry to generate linear oligomeric arrays. The use of optical tweezers also enables the directed deposition and immobilization of supermicelles on surfaces, allowing the precise creation of arrays of soft-matter nano-objects with potentially diverse functionality and a range of applications.

## Abstract (web summary)

Block copolymers can form micelles and assemblies of micelles (supermicelles) when placed in suitable solvents. Here, the authors use optical tweezers to control the arrangement and deposition of supermicelles into higher-order patterned nanostructures.

## Graphical abstract

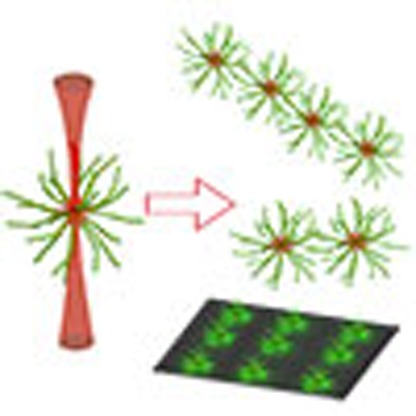

The hierarchical self-assembly of molecular, oligomeric or macromolecular precursors has yielded a wide range of functional nanostructures with precisely controlled dimensions. One specific case that has been the subject of much recent attention involves block copolymers, which consist of two or more distinct polymer segments joined, in most cases, by a covalent linkage. When placed in a solvent that is selective for one of the blocks, these materials undergo self-assembly to form a broad variety of core-shell nanoparticles (micelles). Variations in block ratio, molar mass and experimental conditions, such as temperature, solvent and concentration, have allowed access to a wide range of morphologies, including spheres, cylinders, platelets and other more complex shapes^1^,^2^,^3^. Moreover, in several cases, micelles themselves have been successfully self-assembled into higher order structures or ‘supermicelles'^4^,^5^,^6^,^7^,^8^,^9^.

Although the preparative aspects of micelle and supermicelle formation have recently witnessed a series of recent advances^1^,^2^,^4^,^5^,^6^,^7^,^8^,^9^, the subsequent manipulation of these self-assembled supramolecular structures to create arrays of functionalized soft-matter assemblies represents a key challenge. Despite the success that has been achieved using top-down methods such as flow-based approaches^10^ or lithographic templating using pre-patterned topographic grating patterns for spherical^11^ or cylindrical^12^ micelles, other approaches that provide control over the motion or location of colloidal soft-matter materials are highly desirable.

Optical tweezers, which harness the interaction of light with dielectric objects, have emerged as a useful tool to achieve this end for hard colloidal systems^13^. Highly focused laser beams can be used to trap and subsequently manipulate a range of high refractive index nano/microscale objects including spheres,^14^ rods^15^,^16^ and platelets^17^ with unparalleled control. Although the optical trapping of 1.5–2 μm clusters of 30–40 nm spherical block copolymer micelles has been demonstrated^18^, control over cluster size was not possible, and with stable trapping durations of around 10 s, the ability to manipulate the assemblies was very limited. Recent work on the manipulation of complex materials has included zeolites^19^ and probes created by direct laser writing for optical atomic force microscopy (AFM) techniques^20^,^21^.

The use of a spatial light modulator (SLM) and computer-generated holograms has enabled the generation of hundreds of traps in arbitrary three-dimensional configurations simultaneously. Such techniques have been used for the organization of colloidal materials, a process termed ‘dynamic holographic assembly'^22^. Herein, we describe the use of holographic optical tweezers for the manipulation, deposition and directed assembly of block copolymer (BCP) supermicelle supramolecular assemblies.

## Results

### Preparation of supermicelles

**Figure 1:**
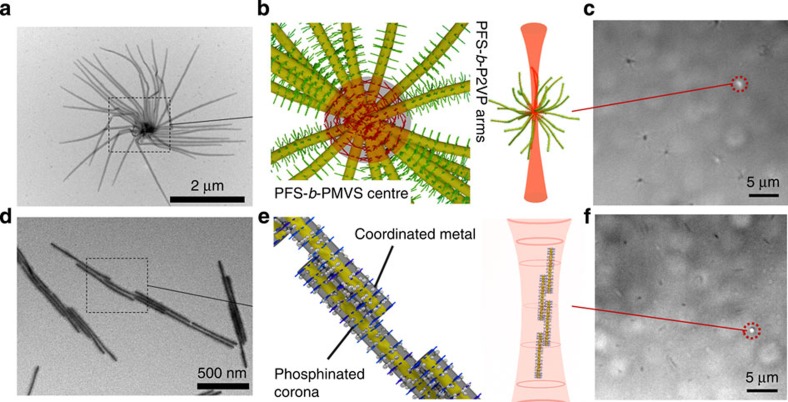
Manipulation of block copolymer nanostructures. (**a**) Transmission electron microscopy (TEM) image of a single spherical supermicelle. (**b**) Schematic showing the structure of a supermicelle's core (PFS: yellow, PMVS: red, P2VP: green). (**c**) Optical microscope image of a trapped supermicelle. (**d**) TEM image of a linear supermicelle formed by coordination-driven self-assembly. (**e**) Schematic showing the structure of linear supermicelle (PFS: yellow, the Pd linkages in phosphinated corona are blue). (**f**) Optical microscopy image showing a trapped linear supermicelle oriented parallel to the beam. The position of the trapping laser beams is indicated by the red dashed rings.

Our work involved two types of soft-matter assemblies. Dandelion-like spherical supermicelles of radius ca 4 μm, fabricated by a previously described method^23^ (Fig. 1a,b) were used for the majority of the experiments. These supermicelles were created by the polar solvent-induced self-assembly of B-A-B amphiphilic triblock comicelles with a crystalline polyferrocenyldimethylsilane (PFS) core and different amorphous coronas, hydrophobic PMVS (polymethylvinylsiloxane) and hydrophilic P2VP (poly(2-vinylpyridine)) in the central and terminal regions, respectively^23^. The amphiphilic comicelle building blocks were prepared by living crystallization-driven self-assembly (CDSA), whereby dissolved PFS-*b*-P2VP block copolymer unimers (that is, molecularly dissolved diblock copolymer) were added to short cylindrical PFS-*b*-PMVS micelles (seeds). During the living CDSA process, the PFS core-forming block of the added unimer crystallizes on the core termini of the seed^24^. Dialysis into iPrOH caused the triblock comicelles to assemble into a spherical morphology, minimizing the contact between the solvent and the central hydrophobic PFS-*b*-PMVS block. Spherical supermicelles with different core diameters (ranging from 100 to 500 nm estimated by transmission electron microscopy (TEM)) and numbers of arms were created by varying the length of the non-polar region of the amphiphilic triblock comicelles. Linear micelle superstructures of length ca 3 μm (Fig. 1d,e) were also studied in several experiments. These were created by the palladium-mediated coordination-driven assembly of cylindrical micelles with a PFS core and phosphinated corona (Lunn D.J., Gould O. E. C., Whittell, G. R., Winnik M. A., Pringle P. G. and Manners I. Coordination-Driven Hierarchical Self-Assembly of Cylindrical Micelles, Manuscript in Preparation). Both the spherical supermicelles and the linear micelle superstructures were sufficiently large (with one dimension of greater than 1 μm) to be detected by optical microscopy.

### Optical manipulation of supermicelles

Initial experiments aimed to demonstrate the manipulation of the spherical supermicelles and the linear superstructures in solution with light. This was facilitated by the high refractive index of the crystalline PFS core of the micelle assemblies^25^, which also enhanced the visualization of the formed nanostructures by optical microscopy (Fig. 1c,f).

When a laser trap was brought close to a spherical supermicelle, which was mobile in solution, the latter was pulled into the trap and held in a stable state in three dimensions. Supermicelles with a range of core diameters and arm lengths were shown to trap. For the linear supermicelles, bringing a laser trap into close proximity caused them to be pulled in and aligned parallel to the laser beam, as has been predicted for dielectric objects of similar dimensions (Supplementary Video 1)^26^.

Linear supermicelles with a range of sizes (approximately 1–3 μm) were also trapped in a stable state in three dimensions. The use of a SLM permitted the creation of multiple traps, and subsequently the simultaneous trapping of multiple spherical supermicelles or linear assemblies.

### Transformation to supermicelle oligomers

**Figure 2:**
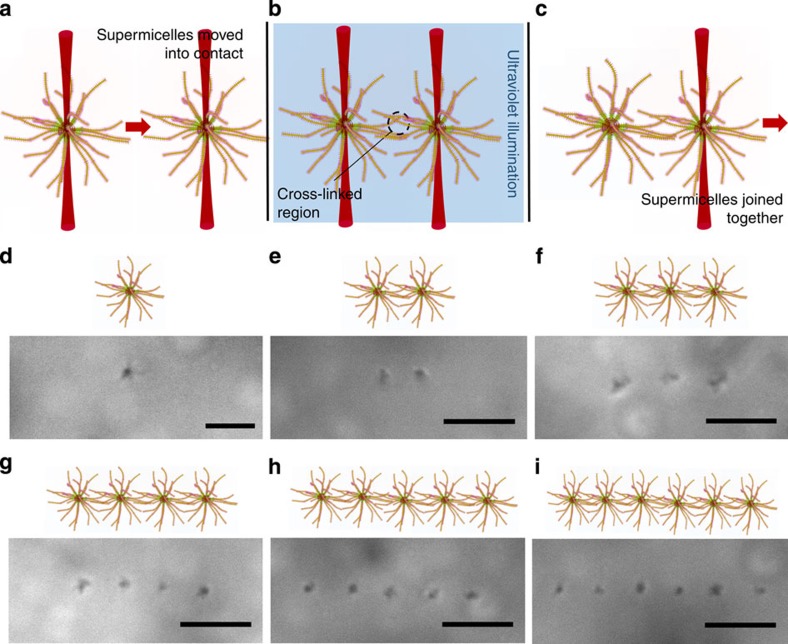
Formation of supermicelle oligomers. (**a**–**c**) Schematic showing assembly process. Two supermicelles were brought into contact using trapping lasers, and ultraviolet illumination was then used to initiate the intermicelle crosslinking. Finally the binding of the supermicelles was confirmed by removing one trapping laser and dragging the combined superstructure. (**d**–**i**) Optical microscope images showing chain-like linear superstructures containing up to six supermicelles in solution. Scale bars are 5 μm.

Previous work has shown that polystyrene latex particles can be bound together by the photogeneration of a polyacrylamide gel in their close proximity^27^. Here, we demonstrate that supermicelles, self-assembled supramolecular assemblies, can be linked together through well-defined coronal chemistry. To link the supermicelles, we used a photoinitiated crosslinking process to bind *in situ* the adjoining coronal chains of two optically trapped supermicelles brought into contact (Fig. 2a–c). For the preparation of suitable structures, supermicelles with cylindrical micelle arms with a poly(methylvinylsiloxane) (PMVS) corona were synthesized (Supplementary Fig. 1). As has been previously reported, ultraviolet light in the presence of a photoinitiator, 2,2-dimethoxy-2-phenylacetophenone (DMPAP), can be used for the crosslinking of the vinyl group of a PMVS containing diblock copolymer^28^. To further verify the crosslinking of micelles with PMVS as the coronal chain ^1^H NMR and TEM studies were performed. Both were consistent with the consumption of vinyl groups on irradiation with a 100-W ultraviolet lamp in the presence of DMPAP (Supplementary Fig. 2). The photoinitiated crosslinking of supermicelles held in an optical trap was enabled by mounting the mercury lamp near the illumination source of the optical setup.

**Figure 3:**
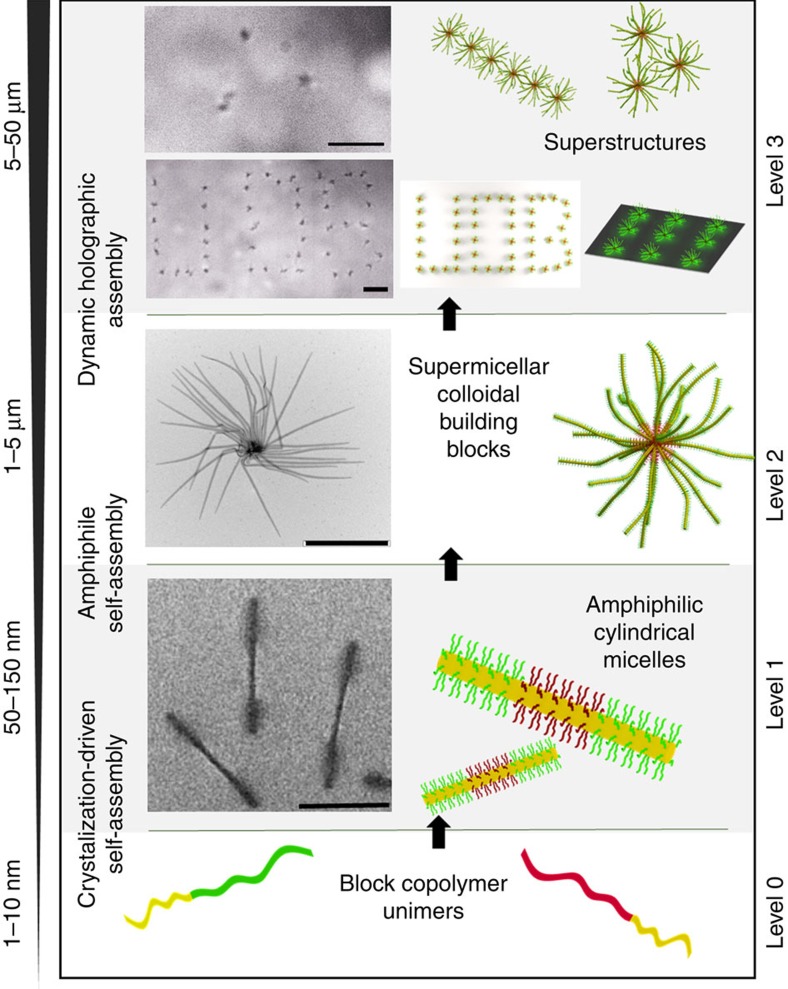
Assembly across multiple hierarchical levels. Level 1: TEM images of amphiphilic triblock cylindrical micelles. Scale bar is 500 nm. Level 2: Supermicelles created by self-assembly of amphiphilic cylindrical micelles. Scale bar is 2 μm. Level 3: Optical microscope images showing supermicellar assemblies created using optical tweezers. Scale bars are 5 μm.

To create an assembly, two (in the simplest case) optically-trapped supermicelles in hexane were brought into contact. The solution, which also contained the photoinitiator, was then irradiated with ultraviolet light, triggering the crosslinking process. After illumination for approximately 30 s, the movements of the supermicelles appeared highly correlated. Following the crosslinking process, the supermicelles were resistant to separation by optical tweezers, allowing one to be dragged by manipulation of the other (Supplementary Fig. 3C and Supplementary Movie 2). Using this photoinduced crosslinking process together with optical tweezers, linear assemblies comprised of up to six supermicelles were fabricated (Fig. 2d–i), as well as two-dimensional triangular superstructures (Fig. 3 Top). The only barrier to the creation of larger oligomeric structures is the output power of the trapping laser, which limits the number of supermicelles that can be trapped simultaneously. Using this approach, we were able to demonstrate the creation of block copolymer nanostructures that bridge four levels of hierarchy—assembly of block copolymers into micelles, comicelles, supermicelles and supermicelle arrays—with unprecedented control at each level (Fig. 3).

### Patterned deposition and growth of supermicelles under flow

The impressive range of complex hierarchical colloidal structures available through solution-phase block copolymer self-assembly possess varied functionality with potential applications as, for example, luminescent nanopixels^29^,^30^, theranostic^9^ and magnetic materials^31^, and templated hybrid nanowires^32^. To facilitate the realization of their full potential, and to also allow their incorporation into devices, a method that allows the controlled deposition of these self-assembled soft-matter structures is highly desirable. We have also found that optical tweezers permit the directed deposition and immobilization of large block copolymer assemblies, allowing the creation of arrays of soft-matter objects. For this work, a purpose-built, adhesive-free flow cell was used, to enable the facile use of a range of solvents (Supplementary Fig. 4B).

**Figure 4:**
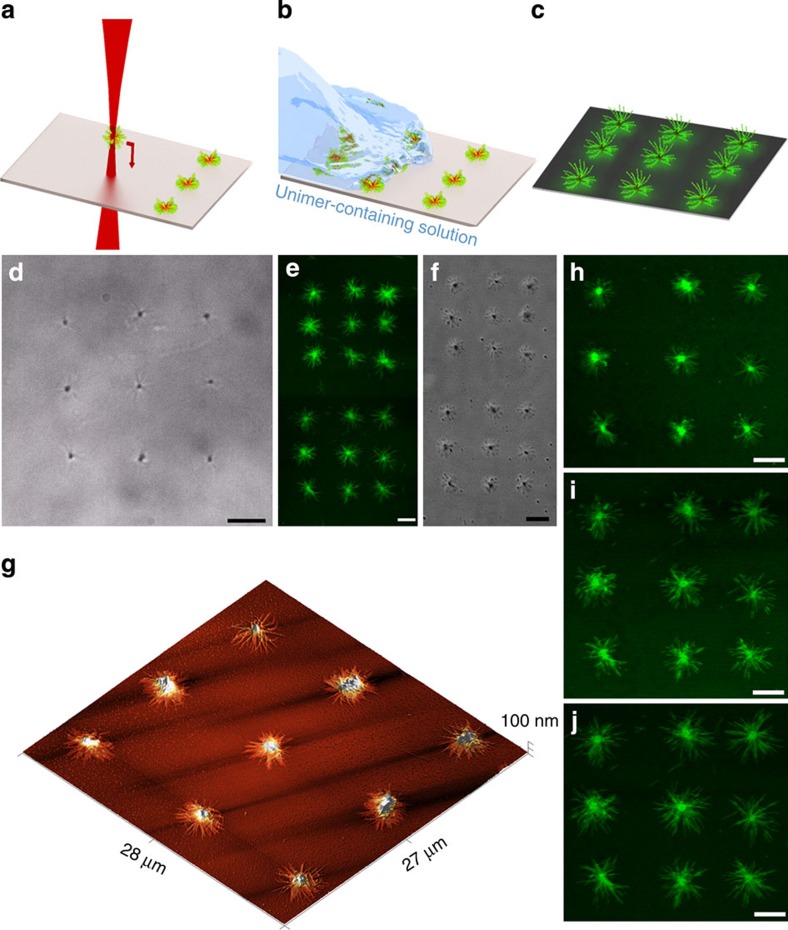
Patterned deposition and growth of supermicelles. Schematics illustrating (**a**) the deposition process, (**b**) the addition of additional unimer in solution and (**c**) the prepared fluorescent arrays. (**d**) Optical bright-field image of a deposited supermicelle array in solution. (**e**) LSCM images of array after addition of solution containing PFS_62_-*b*-(PDMS_605_-r-PMVS_21_) dye functionalized unimer in solution. (**f**) Differential interference contrast image of deposited supermicelle array removed from solution before the addition of unimer. (**g**) High-speed AFM image of a deposited supermicelle array. (**h**–**j**) LSCM images showing the array of supermicelles after addition and removal of 1 (**h**), 2 (**i**) and 3 (**j**) aliquots of a solution containing PFS-*b*-PMVS dye functionalized unimer. Scale bars are 5 μm.

To increase the robustness of the deposition process on the polar glass surface, a polar coronal chain (P2VP) was chosen for the cylindrical micelle arms of a spherical supermicelle. Using the optical tweezers, supermicelles moved axially downward into contact with the glass substrate became immobilized and resistant to further movement. In this way, an array of supermicelles could be rapidly fabricated into arbitrary arrangements. A range of shapes were created, from a basic 3 × 3 array (Fig. 4d) to the letters of the acronym UOB (Fig. 3, TOP). The use of a SLM increased the speed of the process, allowing for multiple supermicelles to be trapped and deposited simultaneously. Characterization of the dried deposited structures with high-speed AFM showed the pronounced crosslinked central region of the supermicelles flanked by cylindrical micelle arms lying flat on the substrate (Fig. 4g).

By connecting a syringe pump to the flow cell, the controlled removal and addition of reagents in solution was also possible. To verify that the termini of the deposited supermicelles remained active to living CDSA, a solution containing a high concentration of PFS-*b*-PMVS unimers was added. A clear increase in size was detected by optical microscopy, where the diameter of the deposited supermicelles increased as more unimer was added.

Further evidence for the growth of the supermicelles was provided by the addition of a PFS-containing block copolymer, PFS_62_-*b*-(PDMS_605_-*r*-PMVS_21_) with a green fluorescent 4,4-difluoro-4-bora-3a,4a-diaza-s-indacen dye grafted onto the coronal chains^30^. A unimer-containing solution of this block copolymer was injected into the flow cell containing the deposited structures to allow further growth. Significantly, the resulting fluorescent supermicelle arrays created in this manner are inaccessible using solution-phase protocols alone as the addition of the non-polar solvent necessary to allow for the growth of the dye-functionalized polymer would cause the uncontrollable aggregation of the polar supermicelles. However, because the supermicelles are immobilized, solvents that would normally cause their aggregation and precipitation can be added, enabling the fabrication of novel structures. Using laser confocal microscopy, the array of immobilized supermicelles was examined before and after the addition of the fluorescent unimer. Before the addition no fluorescence was detected (Supplementary Fig. 5B), whereas shortly after the fluorescent cylindrical micelles were observed to extend from the deposited supermicelles. Through the repeated addition of further unimer, the deposited supermicelles were found to continue to increase in size (Fig. 4h–j). Further characterization (Supplementary Fig. 5C) showed that the growth takes place from the exposed crystal faces found at the centre of the supermicelle and at the ends of the individual cylindrical micelle arms. This approach could, in principle, be extended to allow for the controlled nucleation of any unimer with a sufficient lattice match to the existing crystalline core of the deposited structures^33^.

## Discussion

In summary, we have demonstrated that optical tweezers can be used for the efficient three-dimensional manipulation of supermicelles in solution. Moreover, we describe hierarchical chain-like superstructures that are prepared by binding supermicelles together, demonstrate the patterning of supermicelles into arrays and show that they can be observed to ‘grow' under flow conditions. These two examples illustrate the power of using optical tweezers to not only manipulate self-assembled supramolecular structures, but also to create entirely new superstructures and assemblies through controlled *in situ* chemistry.

In principle, based on the generality of the living CDSA method^34^,^35^,^36^, the work should be extendable to supermicelles based on π-conjugated materials such as polythiophenes. Coupled with the ease of micelle building block functionalization^28^,^30^, it is therefore likely that the approaches described can be extended to a wide variety of chemically and structurally diverse nanoscale objects, opening the door to the fabrication of a range of precisely ordered patterns of supramolecular assemblies with useful electronic and optical properties. The ability to individually place and grow supermicelles with ‘tentacle-like' arms at specific locations on a substrate may enable the creation of nanoscale arrays with applications in microfluidics, catalysis, filtration, sensing and the biosciences.

## Methods

### General considerations

All polymerizations were carried out in an inert atmosphere glovebox. PFS_28_-*b*-PDMS_560_ (*M*_n_=48,200 g mol^−1^, polydispersity index (PDI)=1.02), PFS_55_-*b*-PMVS_825_ (*M*_n_=84,200 g mol^−1^, PDI=1.09), PFS_48_-*b*-P2VP_414_ (*M*_n_=55,100 g mol^−1^, PDI=1.09), PFS_34_-*b*-P2VP_272_ (*M*_n_=36,800 g mol^−1^, PDI=1.07), PFS_20_-*b*-P2VP_140_ (*M*_n_=19,500 g mol^−1^, PDI=1.12), PFS_62_-*b*-(PDMS_605_-r-GreenDye_21_) (PDI=1.21) were synthesized via living anionic ring-opening polymerization (ROP), followed by postpolymerization derivitization in the latter case, as previously reported^30^,^37^,^38^,^39^. Platinum-divinyltetramethyldisiloxane complex in xylenes (Karstedt's Catalyst) with a Pt wt% of 2.1–2.4 was purchased from ABCR. Tetramethyldisiloxane was purchased from Sigma-Aldrich.

### Transmission electron microscopy

Samples for TEM were prepared by drop-casting 5 μl of the micelle solution onto a carbon-coated copper grid, which was placed on a piece of filter paper to remove excess solvent. Bright-field TEM micrographs were obtained on a JEOL1200EX II microscope operating at 120 kV and equipped with an SIS MegaViewIII digital camera.

### Atomic force microscopy

The assembled array of supermicelles was investigated via AFM utilizing a Multi-mode VIII microscope (Bruker) in an ambient environment. Non-resonant PeakForce (Bruker) control was utilized with a ScanAsyst-HR head unit and low stiffness cantilever (SCANASYST-AIR-HR, Bruker), 0.4 N m^−1^, to scan regions up to 100 × 100 μm^2^ in a low force regime <1 nN. The image in Fig. 4 is cropped from an 80 × 80 μm^2^ scan taken at a line rate of 0.78 Hz and a resolution of 5,120 × 5,120 pixels.

### Preparation of spherical supermicelles

Amphiphilic cylindrical B-A-B triblock co-micelles were created by a previously reported method^23^. For the non-polar A block, monodisperse PFS-*b*-PMVS cylindrical micelles were prepared by adding PFS_55_-*b*-PMVS_825_ unimers in tetrahydrofuran (THF) to PFS_28_-*b*-PDMS_560_ crystallites (ca 23 nm) in n-hexanes. To allow for the growth of a polar B block, isopropanol (iPrOH) was added to increase the solution polarity. The 1:3 ratio of hexane to iPrOH allowed for the growth of the polar region without the precipitation of the formed non-polar cylindrical micelles. PFS_48_-*b*-P2VP_414_ unimers in THF were then added to the solution. The formation of amphiphilic cylindrical triblock co-micelles was confirmed by TEM, where the A and B blocks could be clearly distinguished. Dialysis of the solution containing the triblock comicelles against iPrOH yielded supermicellar structures, where the morphology is controlled by varying the ratio of the A and B blocks. For these experiments, an A block length of 260 nm and a B block length of 90 nm were chosen, as they yielded supermicelles with the highest visibility by optical microscopy. Crosslinking of the PMVS central region of the supermicelles was performed by adding 10 μl of tetramethyldisiloxane and 1 μl of Karstedt's catalyst to 1 ml of the dispersed triblock co-micelle solution (in 1:3 hexane/iPrOH) or the corresponding supermicelle solution (in i-PrOH) at room temperature. The mixture was shaken for 10 s and allowed to age for 1 day before TEM and AFM analysis. To increase the diameter of the supermicelle from 500 nm to 5 μm (measured by TEM), additional PFS_48_-*b*-P2VP_414_ or PFS_55_-*b*-PMVS_825_ unimers were added in THF to the solution causing further growth from the exposed termini of the supermicelle, as shown in Supplementary Fig. 1.

### Linear supermicelle preparation by coordination-driven self-assembly

Commercial Pd_2_(dba)_3_ was purified and recrystallized from CHCl_3_ to afford pure Pd_2_(dba)_3_·CHCl_3_ as dark purple/brown crystals. Different amounts of a Pd_2_(dba)_3_·CHCl_3_ stock solution were then added to micelle solutions of cylindrical micelles (*L*_n_=970 nm, *L*_w_=1,005 nm, ^*L*w^/_*L*n_=1.04, *σ*=0.02; 0.5 mg ml^−1^) in EtOAc. This caused the aggregation of the cylindrical micelle precursors into linear fibres surrounded by coordinated palladium (Lunn D. J., Gould O. E. C., Whittell, G. R., Winnik M. A., Pringle P. G. and Manners I. Coordination-Driven Hierarchical Self-Assembly of Cylindrical Micelles. Manuscript in preparation). For these experiments, linear fibres were produced with approximate sizes of 1–3 μm.

### Optical setup

Our system, similar to that described elsewhere^40^, is designed around a commercially available inverted microscope (Axiovert 200, Zeiss). A 3-W 1,064-nm wavelength laser beam is expanded to fill an electrically addressed SLM (P512–0785, Boulder Nonlinear Systems) controlled using a LabVIEW (National Instruments) interface, with each hologram calculation performed on a PC graphics card (nVidia, Quadro FX 5600). The beam is then passed through a polarizing beam splitter and imaged onto the back aperture of an objective lens (numerical aperture=1.3, × 100 Plan-Neufluar, Zeiss). This simultaneously focuses the trapping beam creating the optical traps, and images the sample. Approximately 40% of the laser beam's power is passed by the objective, and is shared between the traps. Movement of the field of view around the sample is achieved with a motorized *x*–*y* stage (MS2000, ASI) and a piezo-electric objective focusing system (Mipos 140 PL, Piezosystem Jena).

### Sample preparation for optical tweezing

To produce a sample for the trapping experiments, a 50-mm borosilicate capillary tube (wall thickness 0.1 mm) was filled with the supermicelle solution. This capillary tube was then placed on a glass microscope slide where a small amount of epoxy adhesive was used to seal it at either end and fix it in place.

### Model studies of supermicelle cross-linking

To verify the crosslinking of spherical supermicelles with a PMVS coronal chain, NMR and TEM studies were performed. Cylindrical micelles were prepared by adding 50 μl of PFS_50_-*b*-PMVS_510_ unimers in THF to 5 ml of hexane causing the formation of cylindrical micelles by homogeneous nucleation. The solutions were then aged for a week to ensure growth was complete. Analysis of the solution by TEM confirmed the formation of micelles with sizes of 5–8 μm. A volume of 100 μl of the photoinitiator DMPAP dissolved in hexane (1 mg ml^−1^) was then added to 1 ml of the micelle solution. This was then placed in a quartz cuvette with high ultraviolet transmission, and exposed to ultraviolet light for 1 h. Micelle solutions were exposed to three different ultraviolet sources: a water-cooled 125-W medium-pressure mercury lamp (mercury lamp A), a desktop 100-W mercury lamp (mercury lamp B) and a handheld 6-W lamp. A 10-μl aliquot was then drop-cast on a carbon-coated copper grid for investigation by TEM (Supplementary Fig. 2A–C). The solvent was removed by evaporation from the remaining solutions, before the micelles were redissolved in dichloromethane, a good solvent for both blocks of the polymer used, for characterization by ^1^H NMR.

When compared with a control sample, the ^1^H NMR spectra of the solutions exposed to mercury lamp A and B indicate a complete reduction in both the amount of vinyl groups and photoinitiator. NMR spectra of the solution irradiated with the handheld lamp indicate a limited reduction in the both the amount of vinyl groups and photoinitiator, consistent with the lamp's lower power output. TEM images of the drop-cast solutions further verified the crosslinking, with large networks of cylindrical micelles present in the samples irradiated with the 100- and 125-W ultraviolet lamp (Supplementary Fig. 2). Some precipitation was also observed in the samples irradiated with the mercury lamp A and B.

### Formation of hierarchical supermicelle oligomers

To produce a sample for the experiments, a 50-mm fused silica capillary tube (wall thickness 0.1 mm) was dipped into the solution containing the supermicelles and photointiator. This capillary tube was then placed on a quartz microscope slide where a small amount of epoxy adhesive was used to seal it at either end and fix it in place. It took around 5 min to manipulate the supermicelles into a desirable position and to perform crosslinking.

### Custom flow cell fabrication

To enable ease of use with a range of solvents, for the deposition experiments an adhesive-free fabrication process known as anodic bonding was used for the cell's preparation^41^,^42^,^43^.

First, a rectangular channel was cut into a 1-mm-thick piece of silicon wafer. Two holes, separated by the length of the channel, were then cut in a 1-mm-thick piece of borosilicate glass chosen for its low coefficient of thermal expansion. This was then positioned on top of the silicon wafer, and both were placed in a box furnace. Graphite electrodes were positioned on top of and underneath the materials, before heating to 500 °C and applying a potential difference of 500 V across the materials. This caused the diffusion of dissociated oxygen ions in the glass to the boundary between the glass and silicon, forming silicon dioxide and generating a strong bond between the two layers.

Polytetrafluoroethylene (PTFE) tubes were then stretched and pulled through the holes until held snugly in place before being trimmed (Supplementary Fig. 4). A thin layer of parafilm was then stretched across the silicon wafer and a 0.1-mm-thick coverslip clamped on top. The device was then heated to 60 °C sealing the coverslip in place, but allowing it to be removed after the deposition of block copolymer (BCP) assemblies.

### Deposition and growth of supermicelle array

A 10-μl solution (0.1 mg ml^−1^) of unimeric dye-functionalized PFS_62_-*b*-(PDMS_605_-*r*-PMVS_21_) in THF was added to hexanes (100 μl) shortly before being injected into the flow cell containing the deposited spherical supermicelles. The solution was kept in contact with the supermicelle array for 5 min, after which time the solution was removed. This process was repeated three times, with the supermicelles displaying an increase in size after each addition. Repeated addition of unimer is necessary as the amount of unimer present in solution is depleted as growth occurs. To allow for easier monitoring of this process, we added repeated aliquots separately, rather than from a continuous feed. This allowed us to stop the growth process while careful characterization (by laser scanning confocal microscopy (LCSM)) was performed. The spatial resolution is limited purely by the size of the supermicelles, as with our method two objects can be placed directly in contact if not on top of each other.

## Additional information

**How to cite this article:** Gould, O. E. C. *et al*. Transformation and patterning of supermicelles using dynamic holographic assembly. *Nat. Commun.* 6:10009 doi: 10.1038/ncomms10009 (2015).

## Supplementary Material

Supplementary Figures Supplementary Figures 1-5

Supplementary Movie 1 Manipulation of linear supermicelle

Supplementary Movie 2 Formation of supermicelle chain
