# Supplementary material for: Transformation and patterning of supermicelles using dynamic holographic assembly
Source: Nat Commun. 2015 Dec 2;6:10009. doi: 10.1038/ncomms10009 (PMC4686664; doi:10.1038/ncomms10009)
Supplement: Supplementary Figures — 1-5 [file ncomms10009-s1.pdf]

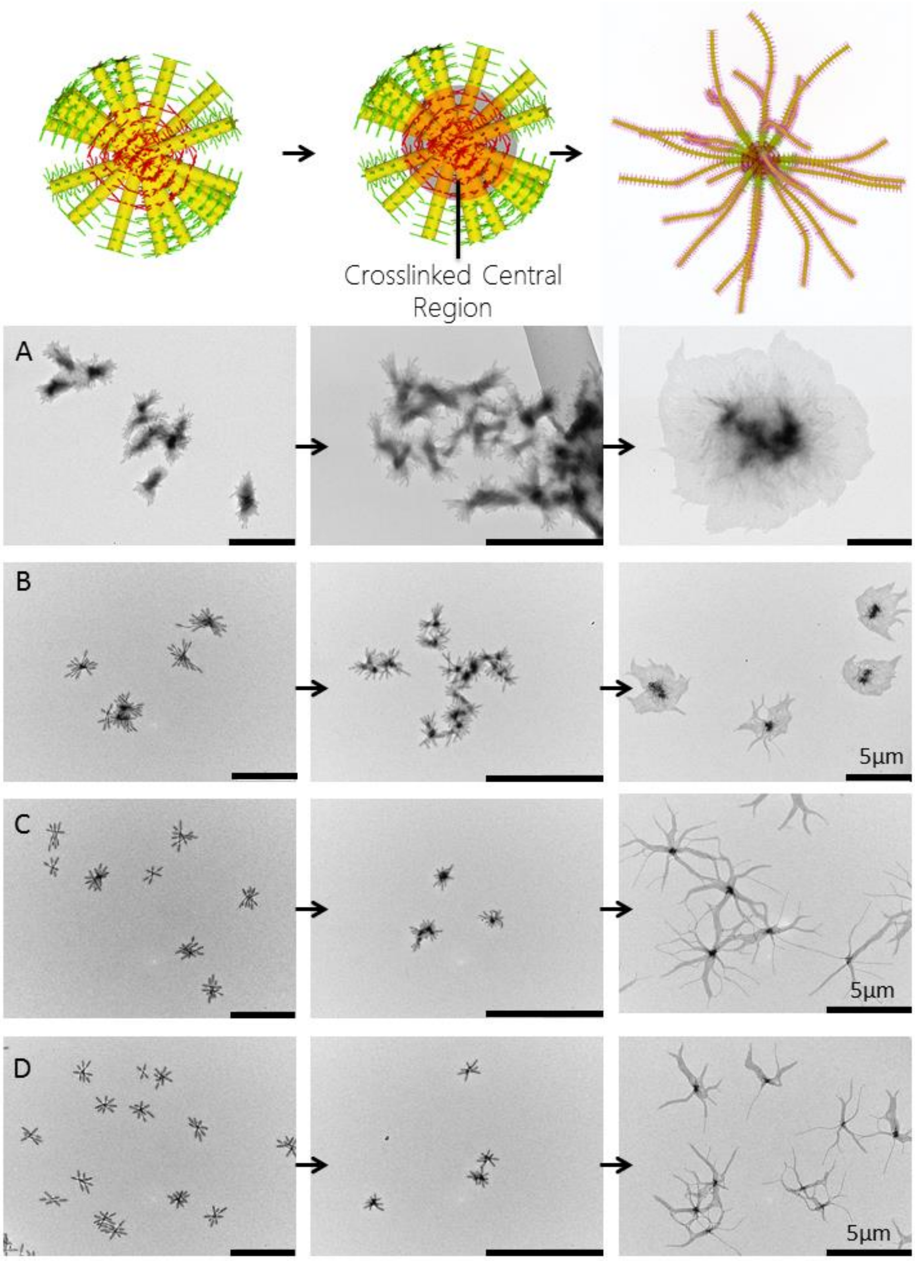

Supplementary Figure 1: Fabrication process for photocrosslinkable supermicelles (PFS yellow, P2VP green) with cylindrical micelle arms with a poly(methylvinylsiloxane) (PMVS) corona (red). **A-D**) TEM images of range of morphologies produced by varying the ratio of the A-B block of the amphiphilic cylindrical micelle seeds. Stage 1 (far left) shows the supermicelles formed after dialysis of amphiphilic cylindrical micelles. Stage 2 (middle) shows the same structures after cross-linking of the central PMVS block. Stage 3 (far right) shows the structures following the addition of PFS_{54}-*b*-$PMVS_{825}$ unimers in THF. Scale bars are 2 µm unless otherwise stated.

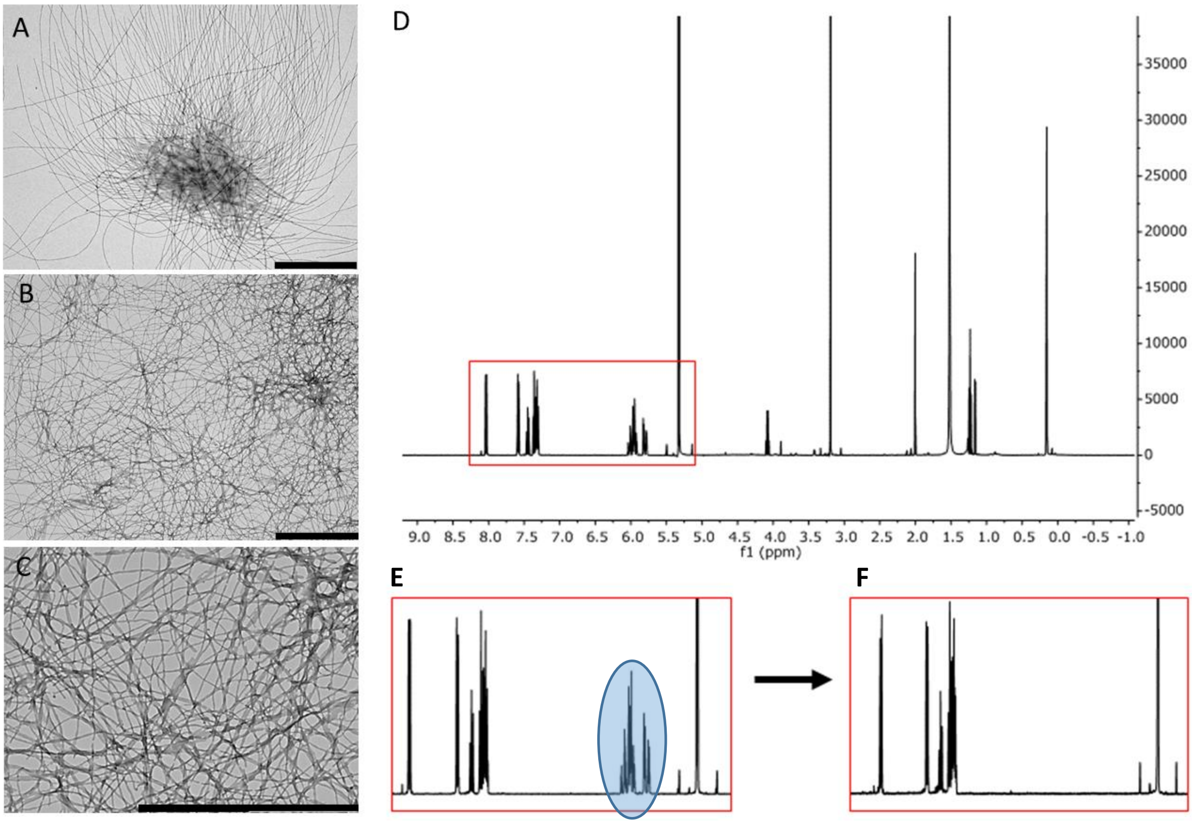

Supplementary Figure 2: TEM images of PFS-*b*-PMVS cylindrical micelles in hexane following addition of DMPA and irradiation with UV light from a 6 W handheld lamp (A) and a 100 W lamp (B&C). (D) ^{1}H NMR spectra (in CD_2Cl_2) before (E) and after (F) exposure to the 100 W mercury lamp used to induce supermicelle binding. The peaks attributed to the vinyl group in the corona of the PFS-*b*-PMVS micelle are marked with a blue circle. Scale bars are 2 µm.

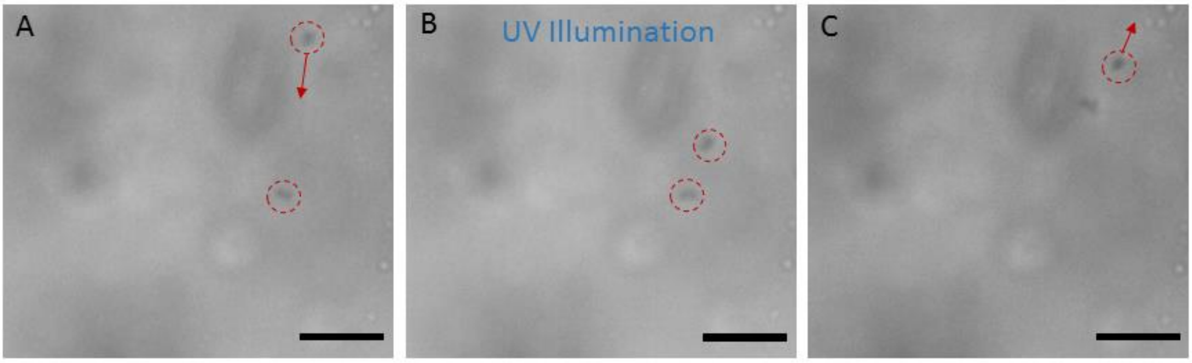

Supplementary Figure 3: Optical microscope images showing: **A)** two trapped supermicelles with PMVS cylindrical micelle arms **B)** the two supermicelles having been brought into contact **C)** the supermicelles following illumination with UV light and crosslinking process, showing one trapped supermicelle and the other untrapped supermicelle being dragged behind it. The red dashed circles mark the positions of the trapping lasers. Scale bars are 5 µm.

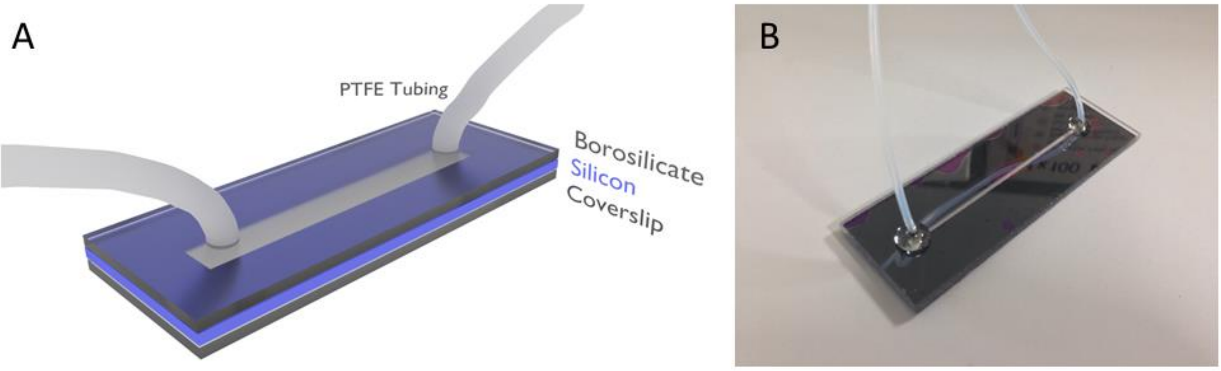

Supplementary Figure 4: A) Schematic of the flow cell fabricated through anodic bonding. B) Photograph of the completed flow cell.

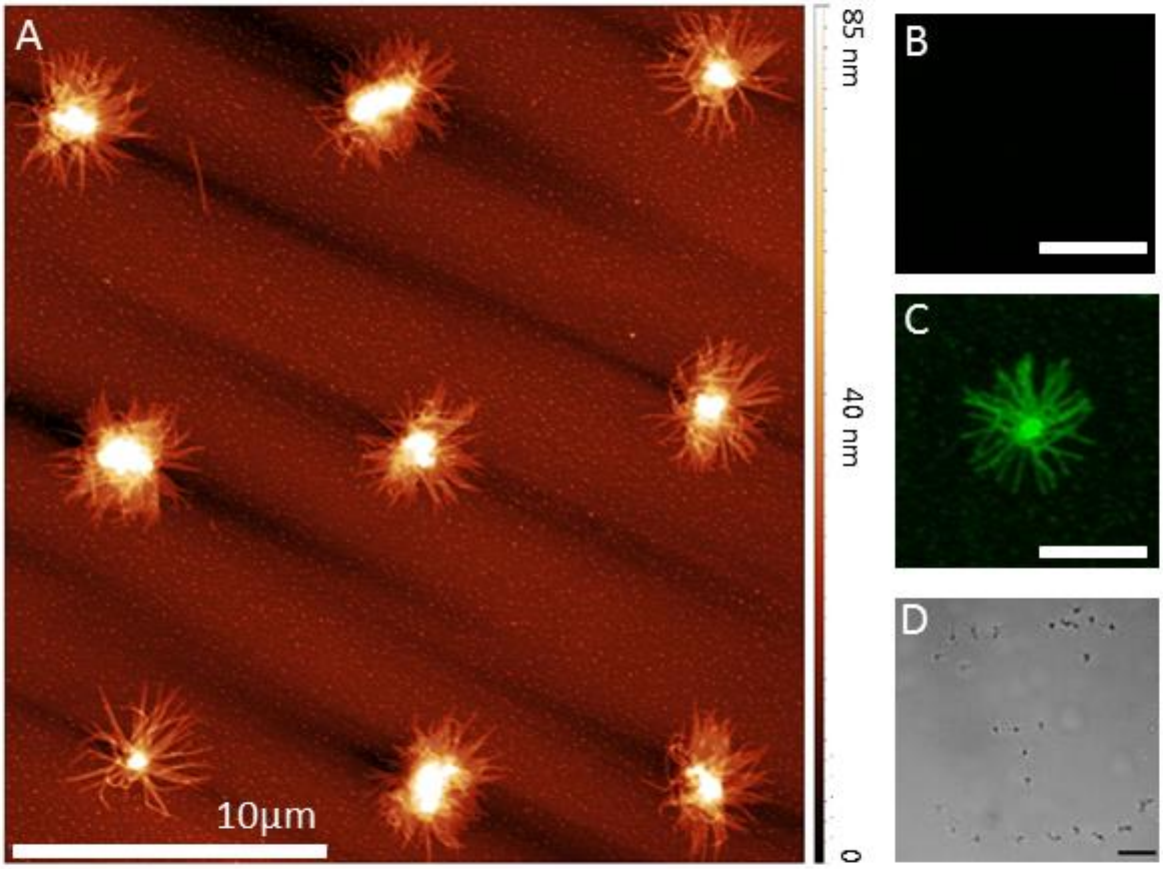

Supplementary Figure 5: **A)** High-Speed AFM image of deposited supermicelle array. **B)** LSCM image of deposited 4 x 4 supermicelle array before fluorescent unimer addition. **C)** Magnified LSCM image of a single deposited supermicelle after fluorescent unimer addition. **D)** Deposited supermicelles in the shape of a smiley face. All scale bars are 5µm unless otherwise stated.
